# Hyperthermia in a pediatric patient with neuroblastoma during anesthesia: a case report

**DOI:** 10.1186/s12893-021-01124-3

**Published:** 2021-03-05

**Authors:** Chuan Wang, Wenqiong Xin, Yi Ji

**Affiliations:** Department of Pediatric Surgery, West China Hospital of Sichuan University, #37 Guo-Xue-Xiang, 610041 Chengdu, China

**Keywords:** Neuroblastoma, Hyperthermia, Catecholamine

## Abstract

**Background:**

Neuroblastoma is the most common malignant extracranial solid tumor in pediatrics patients. Intraoperative hyperthermia is extremely rare in patients with neuroblastoma and can cause a series of complications. Here, we represent a case of neuroblastoma accompanied by hyperthermia during anesthesia, and propose a rational explanation and management options.

**Case presentation:**

The patient had gait disturbance and sitting-related pain without fever. Magnetic resonance imaging revealed a soft tissue mass located in the right posterior mediastinum, paravertebral space and canalis vertebralis. Serum tumor marker screening showed that the patient had increased epinephrine, norepinephrine and neuron specific enolase levels, with an increased 24 hour urine vanillylmandelic acid level. Intraspinal tumor resection was conducted. The temperature of the patient rapidly arose to 40.1 °C over 10 minutes when waiting for tracheal extubation. The arterial gas analysis results indicated malignant hyperthermia was less likely, and dantrolene was not administered. Physical cooling methods were used, and the temperature dropped to 38.6 ℃. The trachea was successfully extubated. Histological results confirmed the diagnosis of neuroblastoma.

**Conclusions:**

Hyperthermia during anesthesia is a serious adverse event. Catecholamines secreted from neuroblatoma cells can lead to hypermetabolism and hyperthermia. Surgeons and anesthesiologists should be aware of the possibility of hyperthermia in patients with neuroblastoma.

## Background

Neuroblastoma originates from neural crest cell derivatives in the sympathetic nervous system. It is the most common malignant extracranial solid tumor and accounts for 8 % of all cancers in pediatrics [1]. Although fever is a common symptom and is present in 26.0 % of pediatric patients with neuroblastoma, intraoperative hyperthermia is extremely rare [2]. Hyperthermia is a serious adverse event, especially during anesthesia. Hyperthermia can cause a series of complications including coagulopathy, increased consumption of oxygen, and neurological damage [3, 4]. To our knowledge, only one study has reported a neuroblastoma patient complicated with intraoperative hyperthermia. The authors did not report any rational explanation or management [5]. In the present report, we present this rare case of neuroblastoma accompanied by hyperthermia during anesthesia, and propose a rational explanation and management.

## Case report

**Fig. 1:**
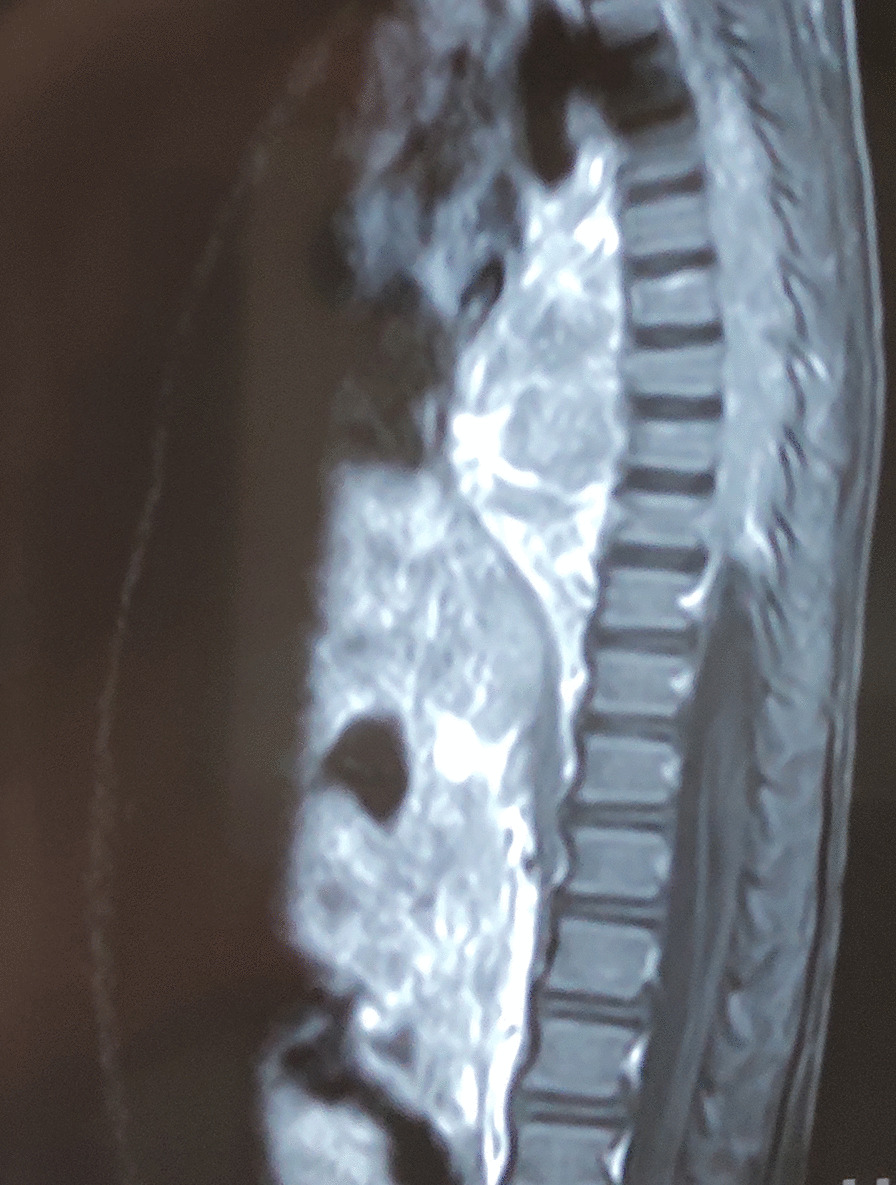
Magnetic resonance imaging showing the tumor was located in right posterior mediastinum, paravertebral space and canalis vertebralis

A 3-year-old Han nationality girl was admitted to our hospital with the chief complaint of gait disturbance and inability to sitting. Additional history included constipation and intermittent abdominal pain without fever. Physical examination found that the muscle strength of the lower limbs was grade II, with normal sensation. Magnetic resonance imaging revealed that a soft tissue mass located in the right posterior mediastinum, paravertebral space and canalis vertebralis that invaded the inferior lobe of the right lung and the 6th to 8th thoracic vertebra (Fig. 1). Single photon emission computed tomography whole-body bone imaging failed to show bone metastasis. No notable abnormality was found in hematological or biochemical tests. Serum tumor marker screening showed that the patient had increased epinephrine (114 ng/L), norepinephrine (17,061 ng/L) and neuron specific enolase (183.30 ng/mL) levels, and a normal alpha fetoprotein level. Laboratory examination revealed that the 24-hour urine vanillylmandelic acid level was 75. 84 µmol/L.

**Fig. 2:**
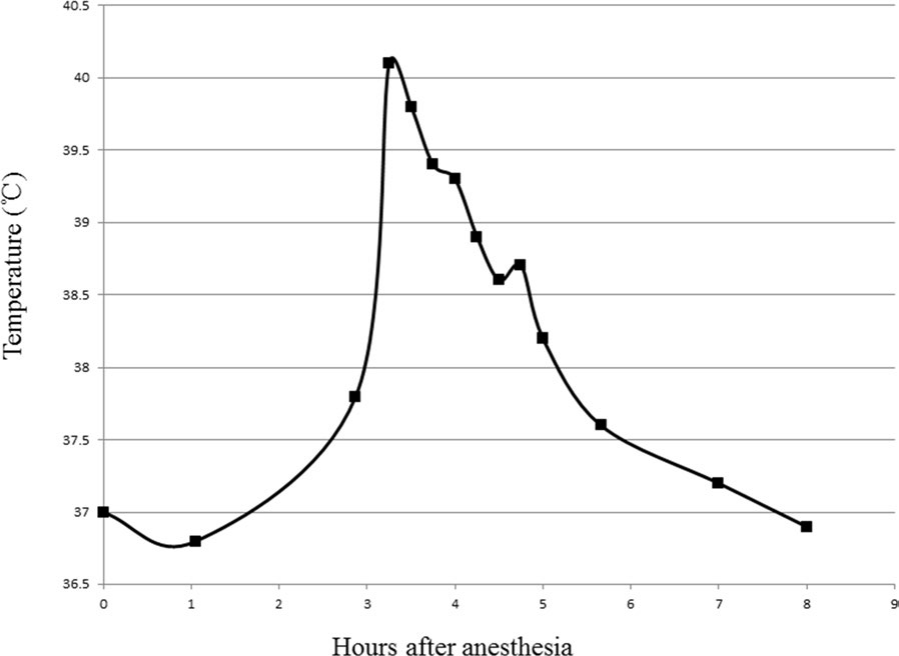
The trend of temperature variation

**Fig. 3:**
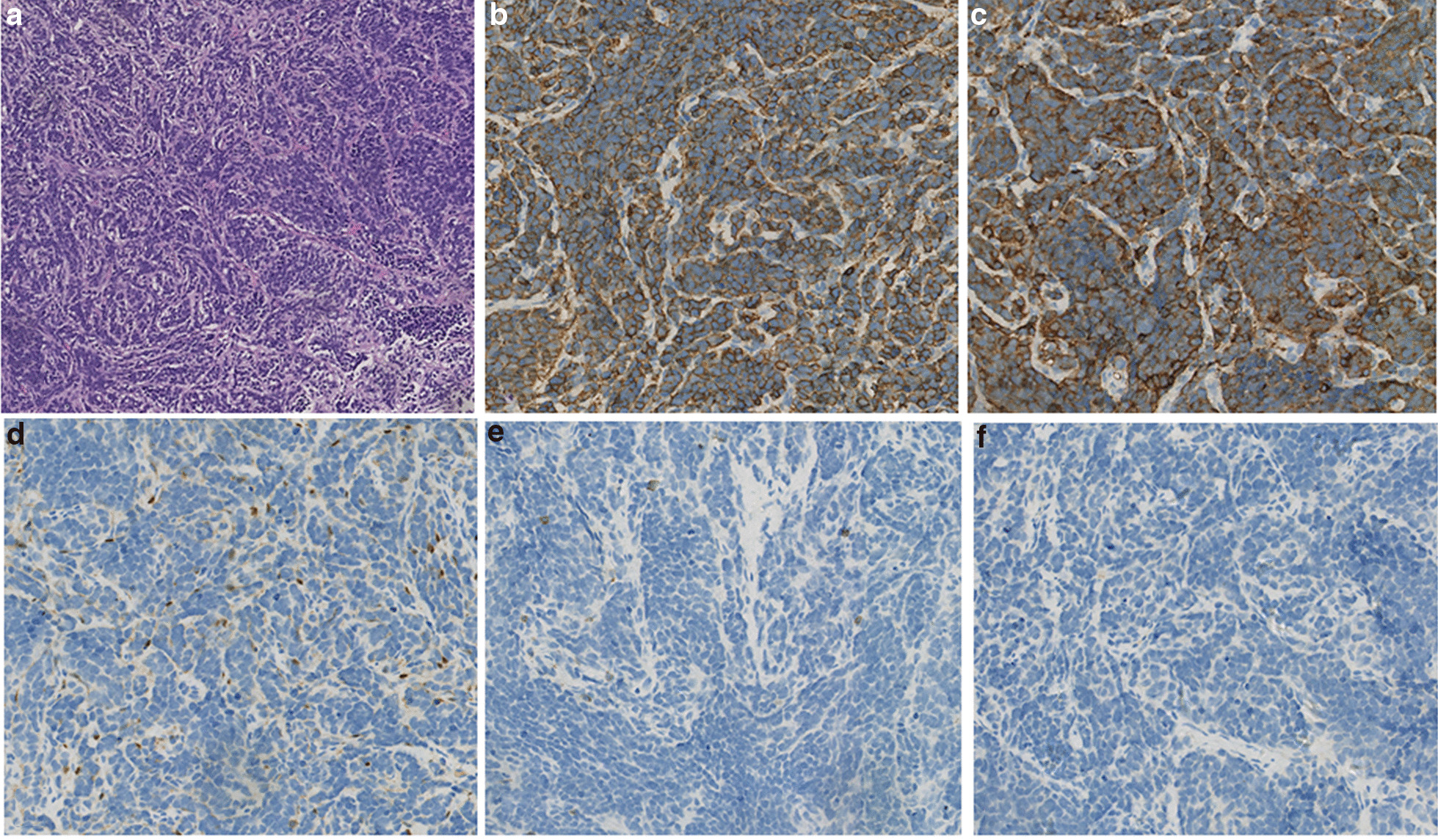
Hematoxylin-eosin staining shows round tumor cells of the same size, with scant cytoplasm and hyperchromatic nuclei, were separated into nest-like structures (**a**) (original magnification 100). Immunohistochemical staining showed that the tumor tissues were positive for CgA (**b**) and Syn (**c**), negative for S-100 (**d**), LCA (**e**) and PCK (**f**) (original magnification 200)

Neuroblastoma was considered as the diagnosis. Intraspinal tumor resection was conducted to relieve the mass effect on the spinal cord and obtain the tumor specimen. Anesthesia was induced with disoprofol combined with sufentanil, atracurium, midazolam and atropine, and then maintained with remifentanil, dexmedetomidine and disoprofol. During the operation, the tumor mass was amputated with the aim of removing the intraspinal tumor tissue, whereas the extraspinal tumor tissue was left. When the patient awaited postoperative tracheal extubation in the operating room, her temperature was 37.8 °C but rapidly increased to 40.1 °C over 10 minutes (Fig. 2). At the same time, her heart rate rose from 146 beats per minute to 152 beats per minute. Ice cap and other physical cooling methods were used. Arterial gas analysis was performed immediately and showed that the values of pH, PaCO_2_ and bicarbonate were within normal ranges. The PaO_2_ was 139.9 mmHg. Because malignant hyperthermia was unlikely to occurr in this situation, dantrolene was not administered. The temperature and heart rate dropped to 38.2 °C and 122 beats per minute within the following 2 hours, respectively. The trachea was successfully extubated when the patient was awake. The patient had intermittent hyperthermia (< 39 °C) during the first two days after operation. Subsequent blood culture and hematological tests showed no evidence of infection. Histological results confirmed the diagnosis of neuroblastoma (Fig. 3).

## Discussion

Intraoperative hyperthermia is an unusual condition in patients with neuroblastoma. Hyperthermia increases oxygen consumption and thrombin generation, impairs endogenous fibrinolysis, and ultimately results in higher risks of perioperative adverse events and worse prognosis [3]. Mayhew et al. reported a case of intraoperative hyperthermia, but they did not explain the reason other than ruling out malignant hyperthermia [5]. In this case, there was a lower chance of malignant hyperthermia because administration of the anesthetic drugs had been discontinued. In addition, arterial blood gas analyses showed that the PaCO_2_ level was within normal ranges, whereas the PaO_2_ level was increased.

Neuroblastoma derives from neural crest cells in the adrenal medulla or in the paraspinal sympathetic ganglia [6]. Increased catecholamine levels occur in 95 % of patients with neuroblastoma. These catecholamines include dopamine, 3-methoxytyramine, homovanillic acid, epinephrine, norepinephrine, normetanephrine, vanillylmandelic acid and metanephrine [7]. Catecholamines can aggravate hypermetabolism and enhance heat production. Gronert et al. found that catecholamines can trigger and promote malignant hyperthermia [8] Experiments have revealed that suppression of catecholamine release can prevent the progression of malignant hyperthermia [9]. In experimental studies, malignant hyperthermia did not occur in hyperthermia-susceptible pigs that had depleted catecholamines before receiving two doses of succinylcholine [9]. This study suggested that catecholamines are strongly associated with the development and progression of hyperthermia. In our case, preoperative serum tumor marker examination showed increased epinephrine and norepinephrine levels, both of which are secreted by the neuroblastoma tumor cells. It is conceivable that the operation can destroy tumor cells and lead to the massive release of catecholamines into the blood. Finally, the patient may develop hypermetabolism and hyperthermia. In this regard, administration of anticatecholamine agents, such as labetalol as a combined alpha- and beta-adrenergic receptor antagonist, may be helpful to lower the temperature. We did not realize that the hyperthermia could have been caused by the release of catecholamines during anesthesia. Thus anticatecholamine agents were not administered.

In conclusion, hyperthermia during anesthesia is a serious adverse event that increases the odds of complications. Surgeons and anesthesiologists should be aware of the possibility of hyperthermia in patients with neuroblastoma. Here we propose an explanation for the appearance of hyperthermia and provide a possible solution.

## Data Availability

The datasets used during the current study are available from the corresponding author on reasonable request.

## References

[1] Li J, Thompson TD, Miller JW, Pollack LA, Stewart SL (2008). Cancer incidence among children and adolescents in the United States, 2001–2003. Pediatrics.

[2] He WG, Yan Y, Tang W, Cai R, Ren G (2017). Clinical and biological features of neuroblastic tumors: A comparison of neuroblastoma and ganglioneuroblastoma. Oncotarget.

[3] Levi M (2018). Hemostasis and thrombosis in extreme temperatures (Hypo- and Hyperthermia). Semin Thromb Hemost.

[4] Walter EJ, Carraretto M (2016). The neurological and cognitive consequences of hyperthermia. Crit Care.

[5] Mayhew JF (2006). Intraoperative hyperthermia in a child with neuroblastoma. Paediatr Anaesth.

[6] Newman EA, Abdessalam S, Aldrink JH, Austin M, Heaton TE, Bruny J (2019). Update on neuroblastoma. J Pediatr Surg.

[7] Verly IRN, Leen R, Meinsma JR, Hooijer GKJ, Savci-Heijink CD, van Nes J (2019). Catecholamine excretion profiles identify clinical subgroups of neuroblastoma patients. Eur J Cancer.

[8] Gronert GA, Theye RA (1976). Halothane-induced porcine malignant hyperthermia: metabolic and hemodynamic changes. Anesthesiology.

[9] Xu X (2019). Magnesium sulfate—an effective agent could delay the progression of fulminant malignant hyperthermia. Med Hypotheses.

